# Development and validation of a predictive model for the risk of symptomatic adjacent segmental degeneration after anterior cervical discectomy and fusion

**DOI:** 10.3389/fneur.2025.1530257

**Published:** 2025-02-17

**Authors:** Xiao Liang, Lijing Ran, Zhenyu Zhang, Xin Xiao, Congyang Wang, Yuwang Du, Hua Jiang

**Affiliations:** ^1^Department of Spine Surgery, Affiliated Hospital of Jining Medical University, Jining, China; ^2^Department of Spine Surgery, The First Affiliated Hospital of Guangxi Medical University, Nanning, China; ^3^Department of Ultrasound, Affiliated Hospital of Jining Medical University, Jining, China

**Keywords:** anterior cervical discectomy and fusion, adjacent segment disease, radiographic parameters, risk factors, prediction model

## Abstract

**Background:**

To investigate the risk factors for symptomatic adjacent segment degeneration (ASD) 5 years after anterior cervical discectomy and fusion (ACDF) and develop and evaluate predictive models.

**Methods:**

A total of 655 patients who underwent ACDF were randomly assigned to the training set (*n* = 393) or validation set (*n* = 262) at a ratio of 6:4. Independent predictors of ASD were selected by LASSO regression and logistic regression analysis. A calibration curve, ROC curve and DCA curve were used to evaluate the model performance.

**Results:**

LASSO regression combined with logistic regression analysis revealed that age, cervical canal stenosis, smaller T1S and smaller cervical lordosis (CL) were risk factors for ASD 5 years after surgery. Nomographic analysis using appeal factors was used to predict the risk of ASD. The area under the ROC curve was 0.711 (95% CI: 0.643–0.780) in the training set and 0.701 (95% CI: 0.618–0.785) in the validation set. The calibration curve showed no significant bias in either set. The DCA indicated that using the nomogram to predict the risk of ASD would be more accurate when the risk threshold probability was 12–53% in the training set and 6–43% in the validation set.

**Conclusion:**

Age, cervical spinal stenosis, a smaller T1S, and a smaller CL are independent risk factors for ASD 5 years after ACDF surgery. Based on these four indicators, we constructed a new clinical prediction model that has a certain predictive effect and is conducive to clinical decision-making and treatment planning.

## Introduction

Anterior cervical discectomy and fusion (ACDF) is the most common surgical procedure for the treatment of cervical spondylopathy radiculopathy and cervical spondylopathy myelopathy; it stabilizes the cervical spine while relieving spinal cord or nerve root compression. A retrospective review of 117 patients with ACDF found that all patients demonstrated clinical improvement as assessed by the JOA (1). However, the occurrence of adjacent segmental degeneration (ASD), including imaging ASD and symptomatic ASD, is often observed during long-term postoperative follow-up (2, 3). Imaging of ASD may progress to symptomatic ASD. The treatment of symptomatic ASD may be appropriate according to the clinical manifestations, and severe pain or neurological dysfunction may require additional surgical intervention (4, 5). ACDF lead to increase forces on adjacent segments causing them to be hypermobility or unstable, which can accelerate the rate of degeneration (6, 7). A study on 497 asymptomatic subjects exploring degenerative changes in cervical intervertebral discs through MR imaging showed that the frequency of all degenerative findings increased linearly with age (8). At present, there is still some controversy about whether ASD represents the surgical complication of cervical spondylosis or the progression of the natural disease course.

A meta-analysis of 83 studies (9) revealed that the prevalence of imaging for ASD after ACDF was 28.28%, the prevalence of symptomatic ASD was 13.34%, and the prevalence of reoperation for ASD was 5.78%. In addition, previous studies have shown that the development of ASD may be influenced by a variety of factors, including patient factors such as age, smoking history, cervical canal stenosis, and pre-existing degenerative changes in adjacent segments, as well as surgical factors such as surgical fusion segments and postoperative cervical sagittal imaging parameters (3, 7, 10–12). Considering the high prevalence rate and potential adverse effects of symptomatic ASD, as well as the complexity of multiple factors causing its occurrence, the aim of this study was to screen the risk factors related to symptomatic ASD through retrospective analysis and construct an effective clinical prediction model to provide certain reference value for predicting the possibility of symptomatic ASD occurrence after ACDF.

## Materials and methods

### Patient selection

Written patient consent was obtained for publication of all aspects of the case including personal and clinical details and images, which may compromise anonymity. This study was approved by the Ethics Committee of the Affiliated Hospital of Jining Medical University (2024-05-C003). This retrospective case-control study was conducted after approval by an institutional review board. A total of 1,064 patients with cervical degenerative disease who underwent ACDF at our hospital between January 2016 and December 2018 were enrolled in this study.

The inclusion criteria were as follows: (1) had cervical spondylotic radiculopathy or cervical spondylotic myelopathy supported by preoperative imaging data and clinical symptoms; (2) were treated with ACDF surgery, and all surgical procedures were performed by two experienced spine surgeons; (3) had surgical levels limited to the range of C3/4, C4/5, C5/6 and C6/7; (4) had complete medical records and demographic information; and (5) had at least 5 years of clinical follow-up.

**Figure 1:**
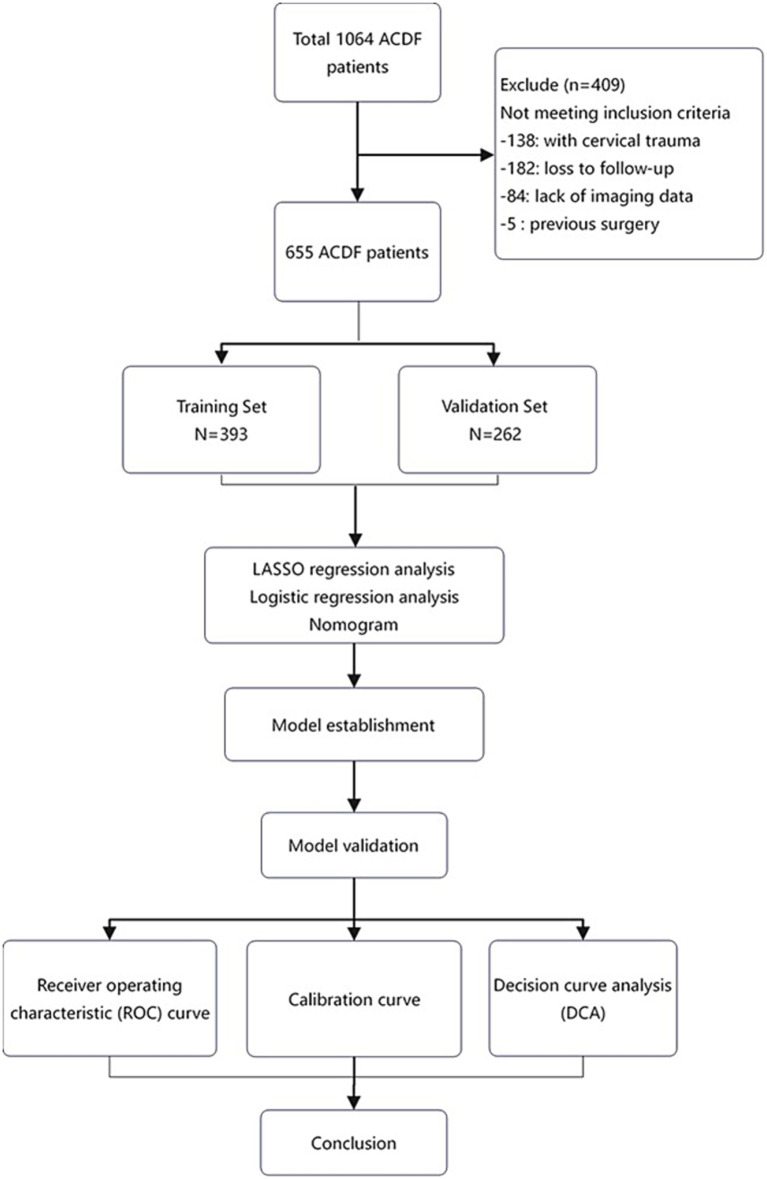
Patient inclusion.

The exclusion criteria were as follows: (1) surgical level C2/3 or C7/T1; (2) history of cervical spine surgery; (3) cervical trauma, infection, scoliosis or tumour; and (4) inability to obtain postoperative follow-up data due to medical or other problems (Figure 1).

### Surgical technique

All patients underwent surgery using the Smith–Robinson anterior approach. After intraoperative cervical discectomy and osteopathic removal, the upper and lower endplates were treated, and nerve root or spinal cord decompression was completed. Subsequently, a cervical titanium cage of appropriate size was inserted into the intervertebral space, and internal fixation was performed using the anterior cervical nail plate system. Postoperative reviews of anterior and lateral cervical radiographs were completed, and patients were usually discharged 5–7 days after surgery, with a collar recommended for 8 weeks after surgery.

### Clinical and radiological evaluation

**Figure 2:**
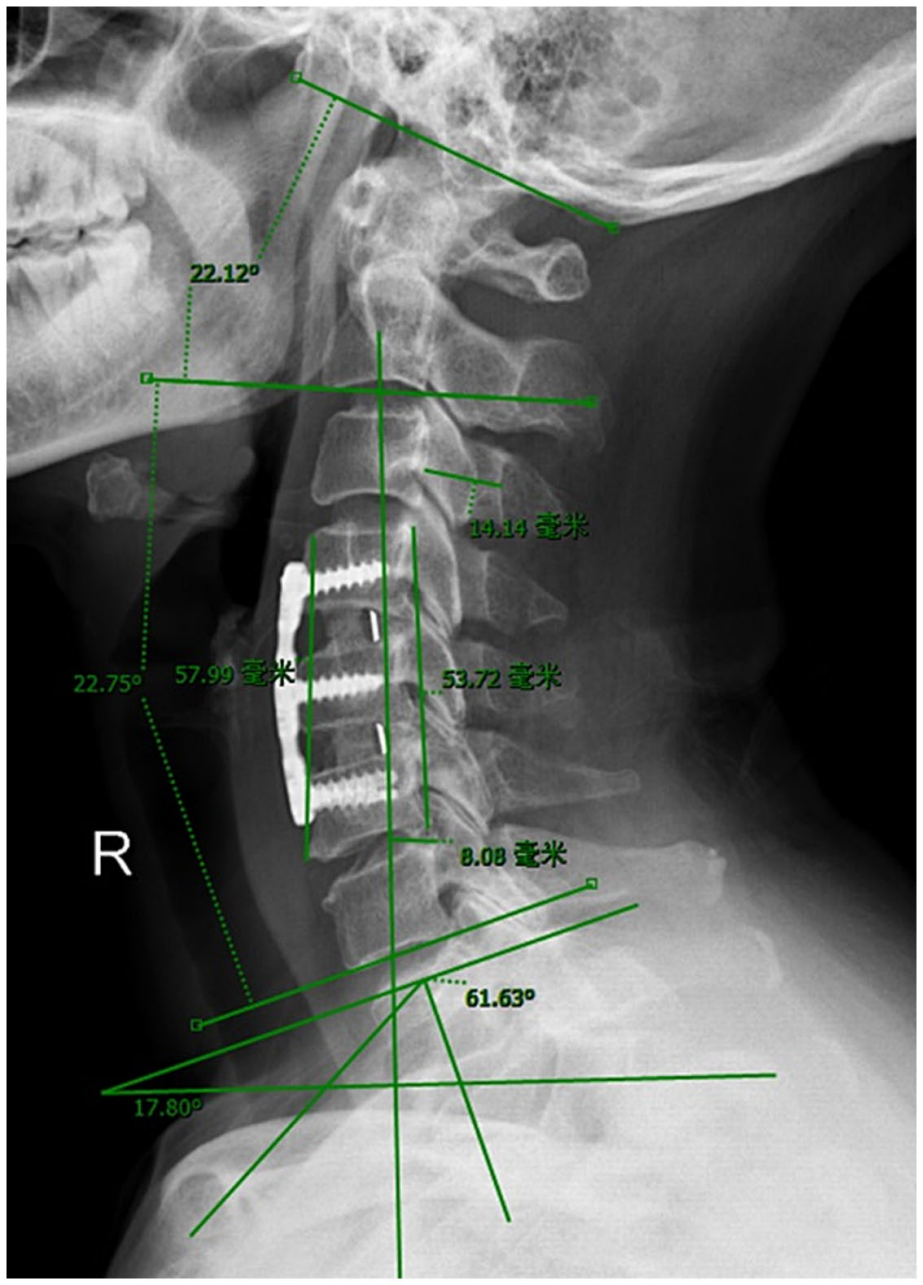
Imaging parameter measurement.

Our study collected clinical information such as sex, age, body mass index (BMI) and duration of symptoms of the enrolled patients. The following radiological variables were measured before discharge: single-level fusion, cervical spinal stenosis, vertebral osteophyte formation, intervertebral distraction (the difference between post-operation and pre-operation in the average height of the anterior and posterior edges of the fusion segment vertebral body), cervical sagittal vertical axis (cSVA), T1 slope (T1S), cervical lordosis (CL), and C0–C2 angle (Figure 2).

### Statistical analysis

The dataset collected from the Affiliated Hospital of Jining Medical University was randomly divided into training and validation sets at a ratio of 6:4, and the variables were compared.

In the training set, the least absolute shrinkage and selection operator (LASSO) method was used to screen the independent risk factors among the candidate risk variables. LASSO constructs a penalty function, gradually compresses the variables, determines the optimal value of the lambda coefficient (*λ*) with the least number of related variables through 10-fold cross-validation, and screens the related predictor variables with *λ*.1se as the tuning parameter. On this basis, multiple logistic regression analysis was used to explore and establish a prediction nomogram for symptomatic ASD. The performance of the nomogram was assessed using receiver operating characteristic (ROC) curves and calibration curves, with the area under the ROC curve (AUC) ranging from 0.5 (not discriminant) to 1 (completely discriminant). Calibration lines and deviations above or below the 45-degree diagonal reflect underprediction or overprediction, respectively. Decision curve analysis (DCA) was also performed to determine the net benefit threshold of prediction. All the statistical analyses were performed using R software (version 4.2.2; R Foundation for Statistical Computing, Vienna, Austria).

## Results

### Demographic and clinical characteristics

In this study, 1,064 patients who underwent ACDF were screened, and a total of 655 patients were included. According to the follow-up results, 122 patients were classified as having symptomatic ASD, among whom 19 patients improved after the second revision surgery, and the remaining 103 patients achieved the ideal therapeutic effect through conservative treatment. The entire set of 655 patients was assigned to the training set (*n* = 393) or the validation set (*n* = 262). There were 77 (19.6%) and 45 (17.2%) symptomatic ASD patients in the training set and the validation set, respectively, with no significant difference. In addition, no significant differences were observed regarding the baseline demographic or clinical characteristics between the two groups (Table 1).

**Table 1:** Demographic characteristics of the training set and validation set.

Characteristic	Entire set		*p*-value^b^
	Training set, *N* = 393^a^	Validation set, *N* = 262^a^	
Sex			0.423
Female	185 (47.1%)	115 (43.9%)	
Male	208 (52.9%)	147 (56.1%)	
Age (>50 years)			0.273
No	176 (44.8%)	106 (40.5%)	
Yes	217 (55.2%)	156 (59.5%)	
BMI (>24)			0.514
No	233 (59.3%)	162 (61.8%)	
Yes	160 (40.7%)	100 (38.2%)	
Duration of symptoms (>1 year)			0.968
No	313 (79.6%)	209 (79.8%)	
Yes	80 (20.4%)	53 (20.2%)	
Single-level fusion			0.814
No	260 (66.2%)	171 (65.3%)	
Yes	133 (33.8%)	91 (34.7%)	
Cervical spinal stenosis			0.524
No	253 (64.4%)	175 (66.8%)	
Yes	140 (35.6%)	87 (33.2%)	
Vertebral osteophyte formation			0.761
No	264 (67.2%)	173 (66.0%)	
Yes	129 (32.8%)	89 (34.0%)	
Intervertebral distraction (>3 mm)			0.592
No	253 (64.4%)	174 (66.4%)	
Yes	140 (35.6%)	88 (33.6%)	
cSVA (>20 mm)			0.723
No	224 (57.0%)	153 (58.4%)	
Yes	169 (43.0%)	109 (41.6%)	
T1S (>20°)			0.678
No	194 (49.4%)	125 (47.7%)	
Yes	199 (50.6%)	137 (52.3%)	
CL (>15°)			0.405
No	220 (56.0%)	138 (52.7%)	
Yes	173 (44.0%)	124 (47.3%)	
C0–C2 angle (°) (>30°)			0.603
No	236 (60.1%)	152 (58.0%)	
Yes	157 (39.9%)	110 (42.0%)	

a *n* (%).

b Pearson’s chi-squared test.

### Variable selection

**Figure 3:**
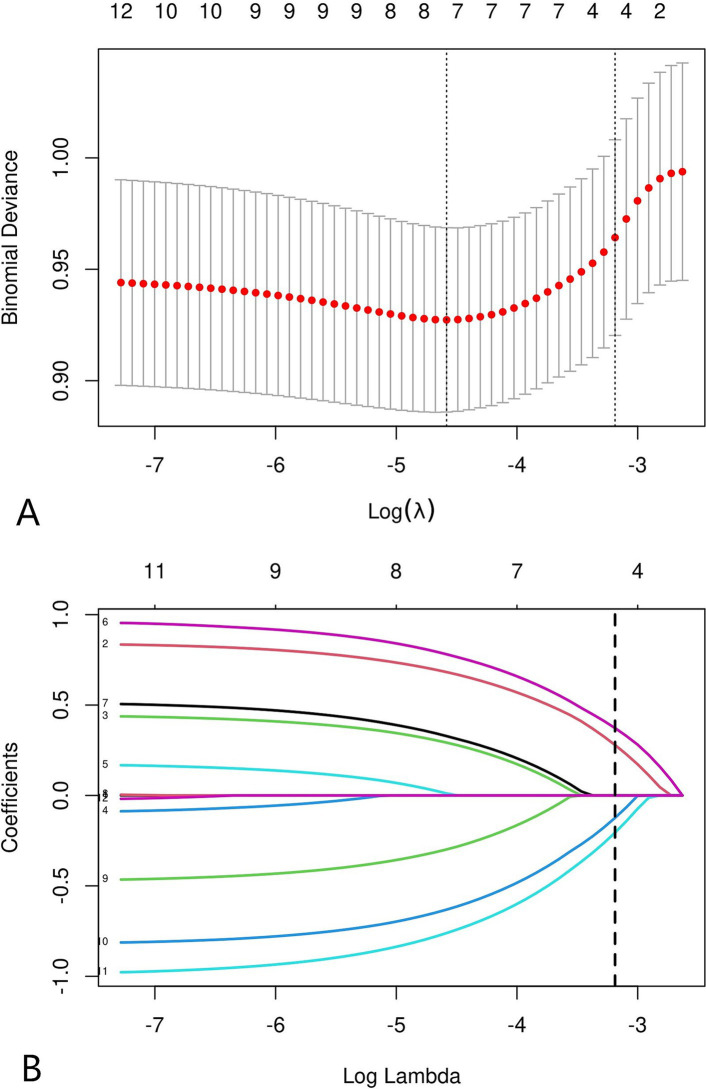
LASSO coefficient distribution of 12 risk factors **(A)**. The four risk factors selected by LASSO regression analysis included age, cervical spinal stenosis, T1S, CL **(B)**.

All variables (including sex, age, BMI, duration of symptoms, single-level fusion, cervical spinal stenosis, vertebral osteophyte formation, intervertebral distraction, cSVA, T1S, CL, and C0–C2 angle) were screened by LASSO regression (Figure 3). Age, cervical spinal stenosis, T1S, and CL were selected as potential predictors, and univariate and multivariate logistic regression were subsequently used to analyse the selected factors. Age, cervical spinal stenosis, T1S, and CL were found to be independent predictors of symptomatic ASD after ACDF (Table 2).

**Table 2:** Results of univariate and multivariate logistic regression analysis for training set.

Characteristic	Univariate logistic regression analysis			Multivariate logistic regression analysis		
	OR	95% CI	*p*-value	OR	95% CI	*p*-value
Age (>50 years)						
No	—	—		—	—	
Yes	2.38	1.38, 4.08	0.002	2.41	1.38, 4.23	0.002
Cervical spinal stenosis						
No	—	—		—	—	
Yes	2.50	1.50, 4.14	<0.001	2.36	1.40, 4.00	0.001
T1S (>20°)						
No	—	—		—	—	
Yes	0.52	0.31, 0.87	0.012	0.46	0.26, 0.79	0.005
CL (>15°)						
No	—	—		—	—	
Yes	0.47	0.28, 0.81	0.006	0.39	0.22, 0.69	0.001

### Nomogram prediction model construction

**Figure 4:**
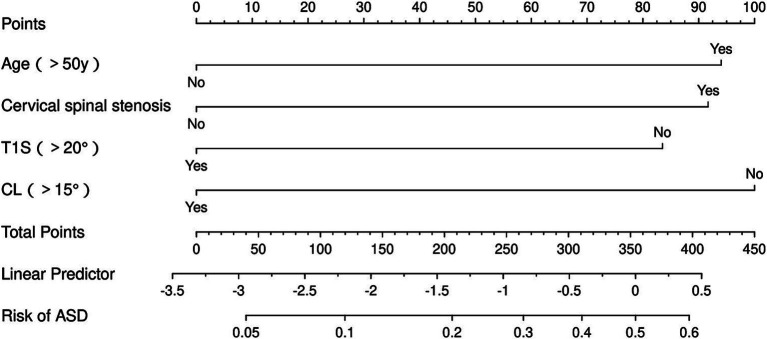
Nomogram plot prediction model of ASD after ACDF.

Based on the selected predictive factors (age, cervical spinal stenosis, T1S stage, and CL status), a nomogram was constructed. The position of each risk factor on the column chart was used to obtain a separate score for that factor, and the scores for all risk factors were added to obtain an overall score. The total corresponding probability value represents the likelihood of developing symptomatic ASD in the corresponding ACDF patient 5 years after surgery (Figure 4).

### Validation of the nomogram prediction model

**Figure 5:**
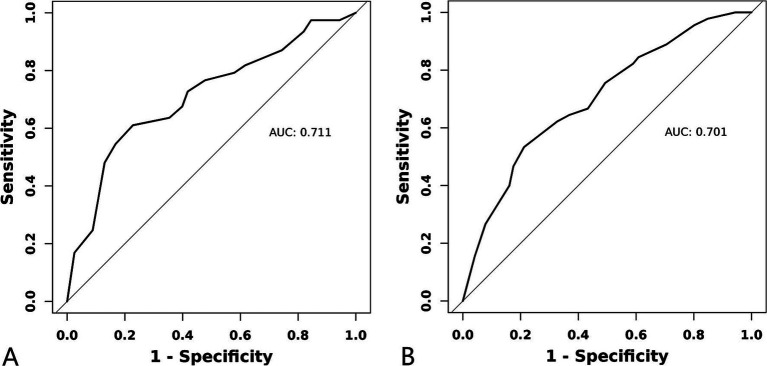
ROC curve of a clinical prediction model for ASD. **(A)** Training set. **(B)** Validation set.

**Figure 6:**
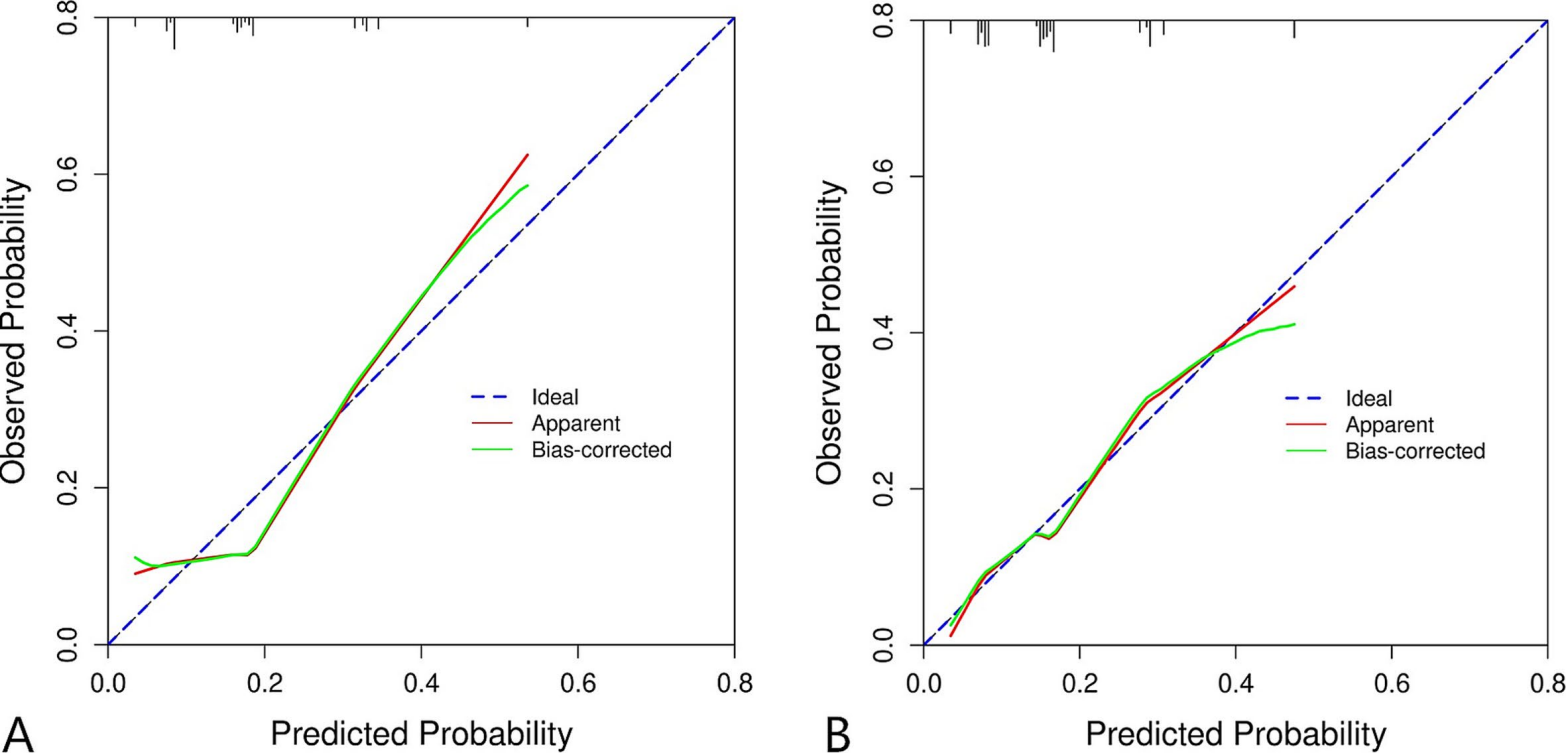
Calibration curve of clinical prediction model for ASD. **(A)** Training set. **(B)** Validation set.

**Figure 7:**
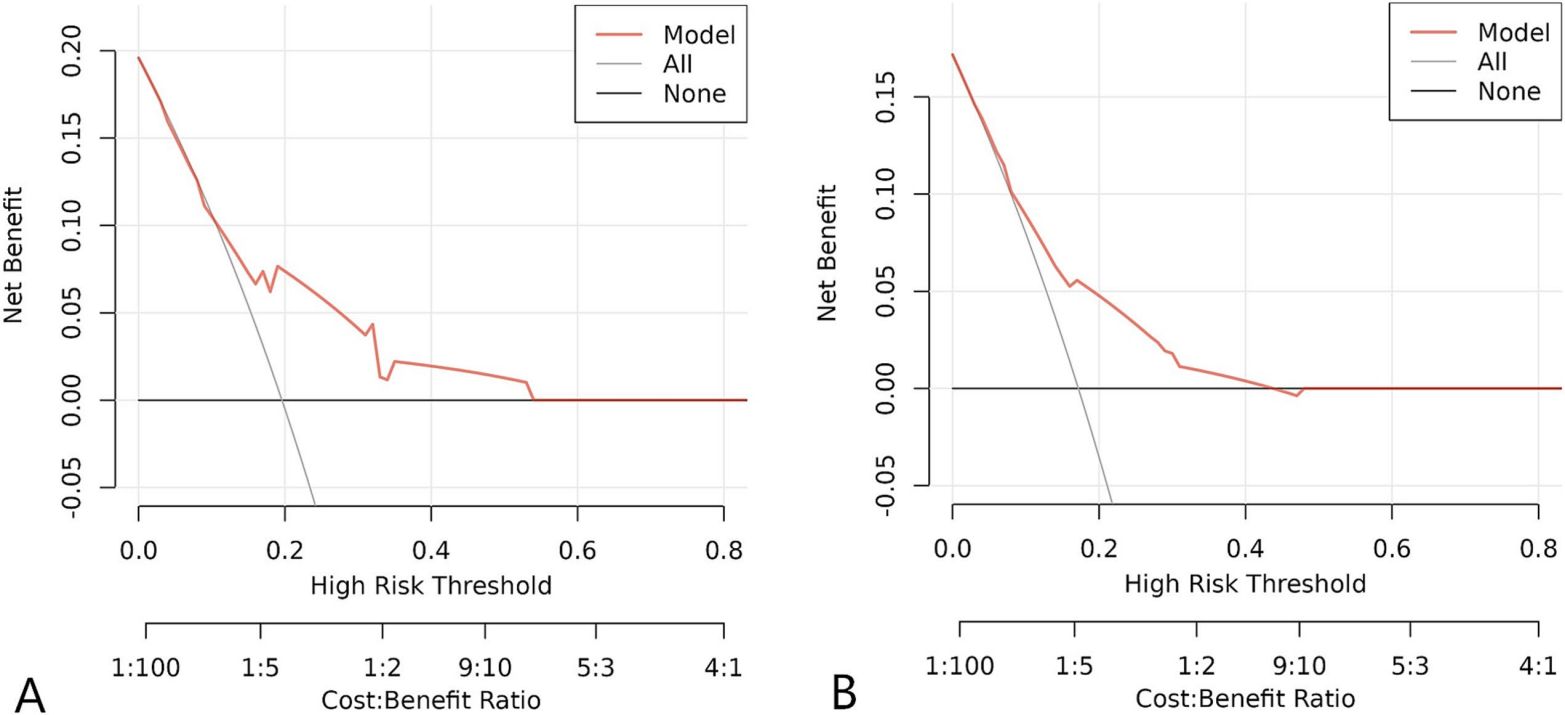
DCA curve of the clinical prediction model of ASD. **(A)** Training set. **(B)** Validation set.

For the training set, the area under the ROC curve was 0.711 (95% CI: 0.643 to 0.780), indicating that the model has a certain discrimination ability, while the area under the ROC curve of the verification set was 0.701 (95% CI: 0.618 to 0.785), indicating acceptable discrimination ability (Figures 5A,B). The calibration curve demonstrated a good correlation between the observed and predicted probabilities in both the training set and the validation set, which indicates that the predicted results were consistent with the actual findings (Figures 6A,B). The DCA results showed that when the risk threshold probability was 12–53% in the training set and 6–43% in the validation set, it was more accurate to predict the risk of symptomatic ASD occurrence using the nomogram (Figures 7A,B).

## Discussion

ACDF is an effective surgical method for treating cervical degenerative diseases. However, potential complications such as ASD may occur after surgery. ASD is a disease characterized by degenerative changes in spinal segments adjacent to fusion levels that can lead to symptoms such as neck pain, radiculopathy or myelopathy (13). The incidence of symptomatic ASD was defined as the percentage of asymptomatic patients who developed new disease during a given follow-up period (12). A 10-year follow-up study (14) showed that imaging and clinical ASD were found in 92.1 and 19.2% of patients, respectively. However, in our study, not all patients with symptomatic proximal spondylosis were treated with a second surgery, and most patients achieved significant symptom relief through medication and rehabilitation therapy, similar to those with primary disease. A meta-analysis of 83 studies (9) revealed that the prevalence of radiological ASD, symptomatic ASD, and reoperative ASD after cervical surgery was 28.28, 13.34, and 5.78%, respectively. Other studies have also shown that 90% of patients who develop symptoms and require MR evaluation do not need to undergo surgery again (11, 12). In our research, the reoperation rate after ACDF was 2.9% (19/655), which was close to the results of other studies (15, 16).

Previous studies have confirmed that the development of ASD may be related to age, BMI, genetic factors, sagittal imaging parameters, number of surgeries, and heterotopic ossification (15, 17–20). Advanced age is an important risk factor for symptomatic ASD in our study, be consistent with it, Li et al. (21) found that older patients are more likely to develop cervical kyphosis after ACDF. However, some studies have shown that there is no significant association between age and the need for reoperation for symptomatic ASD (22). Shahzad et al. (15) reported the highest overall incidence of reoperation secondary to symptomatic ASD in the 30–39-year-old age group. In the current literature, there is mixed consensus on whether age plays an important role in the development of ASD that necessitates surgical intervention (15, 20–23). One study revealed that while older patients are more likely to develop cervical ASD, in fact, young patients often choose to undergo reoperation after the onset of symptoms in order to pursue better neck function, whereas older patients often have other diseases that make them unsuitable for surgery and thus have a lower revision rate than younger patients (2). Based on the above description, we recommend nonfusion decompression in younger patients and extended surgery in older patients. For older patients, when signs of degeneration are found in adjacent segments, even if no symptoms appear, we also recommend that clinicians consider expanding the surgical scope to avoid secondary revision of adjacent segments.

Many previous studies and meta-analyses have focused on imaging parameters as possible risk factors for the development of ASD after ACDF (2, 7, 10). Similarly, our study confirmed that cervical spinal stenosis, T1S and CL are predictors of ASD. Developmental cervical spinal stenosis was reported as an important risk factor for ASD after ACDF, and an anteroposterior cervical canal diameter of 13.0 mm can be used as a threshold for predicting imaging ASD (24). Morishita et al. (25) found that there were statistically significant differences in the pathological and kinematic characteristics of cervical vertebrae with cervical canal diameters less than 13 mm and greater than 13 mm, and it was believed that the mechanical load of cervical vertebrae may increase in patients with cervical spinal stenosis due to their unique kinematic characteristics, which may greatly promote pathological changes in cervical disc degeneration. For patients with spinal stenosis, posterior cervical canal augmentation might be a better surgical option.

The natural curvature of the cervical spine maintains a lordotic shape to compensate for the kyphotic curvature of the thoracic spine. Numerous clinical studies have shown a significant positive correlation between T1S and CL (26–28). T1S is an indicator of sagittal balance of the T1 vertebral body. As the cervical spine is based on the upper endplate of T1, changes in its angle greatly affect the balance of the entire cervical spine. When T1S increases, the center of gravity of the head shifts forward and downward. In order to maintain visual level, compensatory increase in CL is required to maintain sagittal balance of the cervical spine. For many surgeons, the improvement and preservation of cervical lordosis is a key goal of ACDF surgery, but it is also debatable (29–31). Some studies indicated that patients can achieve long-term and satisfactory recovery of clinical function regardless of whether the CL improves (31). Surgeons can use the anterior screw-plate system to correct cervical kyphosis, but there is no consensus on the optimal threshold for CL (30). However, patients with ASD had significantly lower postoperative cervical lordosis than patients without ASD on the meta-analysis performed on two studies (32, 33), which is consistent with our findings. For patients with smaller CL, we advocate using large fusion as much as possible during the surgical process to restore the height of the spinal gap and increase cervical lordosis.

T1S and TIA have a significant effect on the sagittal balance of the cervical spine because the upper endplate of T1 is the base of the cervical spine. It has been found that in asymptomatic individuals, those with larger T1S require greater cervical lordosis to maintain the physiological sagittal balance of the cervical spine (34). However, with increasing age, the head shows an overall forward tendency, resulting in a loss of balance on the sagittal surface of the cervical spine. To maintain forward gaze leading to an increase in cervical lordosis, the T1 at the base of the neck becomes more horizontal to allow for this lordosis, which is a compensatory mechanism proposed by the researcher (35). Cervical surgery artificially changes the physiological curvature of the cervical spine, but the constancy of the T1S and TIA leads to abnormal force conduction and accelerates cervical degeneration (19). In general, the cervical spine tends to recover from a small curvature to a normal physiological curvature after ACDF surgery, and a small T1S cannot adapt to stress changes in the entire cervical spine. In patients with small T1S, the cervical spine will be subjected to greater vertical pressure, which will accelerate cervical disc degeneration. When the cervical spine maintains its natural lordotic curvature, the load on the head is distributed primarily in the posterior column of the cervical spine (approximately 64%) (28). As cervical lordosis decreases, the moment, instantaneous axis of rotation (IAR), and lever arm length applied to the cervical spine may change. The load on the anterior cervical column increases, and if not stopped in time, the kyphosis will continue to progress, the posterior annulus fibrosus will separate from the endplates, and reactive osteogenesis will occur at the fibrous separation, and these excess bony remnants can encroach posteriorly into the spinal canal and compress tissues such as the spinal cord, nerves, and blood vessels. The intervertebral disc adjacent to the surgical segment is the stress concentration site, which is more prone to disc degeneration (19). The same conclusion is also drawn on the impact of TIA on ASD, which is consistent with the positive correlation between TIA and T1S (36). However, TIA was not included as a candidate impact factor in this study due to the sample size.

In this study, we used ACDF surgical data from our hospital to analyse the independent risk factors for ASD and successfully constructed a clinical prediction model. However, through the verification and evaluation of this prediction model, it can be found that there are still some limitations in this study. The relatively low AUC values of the ROC curve of the model indicates that the prediction accuracy of the model is limited, and the results of the DCA curve also show that the model can only generate a high net benefit for the prediction under the limited risk threshold probability. Due to the limited sample size, there may be some potential confounding factors not included in this model, which will have a certain impact on the accuracy of this model. In addition, this study was conducted on a patient-based basis in our unit and may not be representative of the broader population, and external validation in different populations is essential for the generality of our findings.

## Conclusion

This study revealed that age, cervical spinal stenosis, a smaller T1S, and a smaller CL were associated with an increased risk of symptomatic ASD after ACDF. A clinical prediction model based on these four factors might be a useful risk stratification indicator for ASD and could be beneficial for surgeons in the selection of fusion segments for primary surgery.

## Data Availability

The original contributions presented in the study are included in the article/supplementary material, further inquiries can be directed to the corresponding author.
